# Use of the melanoma vaccine in 38 dogs: The South African experience

**DOI:** 10.4102/jsava.v86i1.1246

**Published:** 2015-04-30

**Authors:** Joanne L. McLean, Remo G. Lobetti

**Affiliations:** 1 Bryanston Veterinary Hospital, Bryanston, South Africa

## Abstract

The commercially available vaccine Oncept^®^ is indicated for the management of dogs with stage II or III oral melanoma after local control has been achieved. Survival times in dogs with both oral and digit melanoma have been shown to be significantly increased following vaccination. This retrospective study was designed to document the investigators’ experiences with Oncept^®^ vaccine when used as an adjunct therapy for treatment of stage II–IV oral, digit and malignant melanoma of other sites after local control had been achieved in dogs presented to a South African specialist referral veterinary practice. Thirty-eight dogs diagnosed with melanoma (25 oral, 6 digit and 7 infiltrative at various other sites) underwent a combination of surgical excision and Oncept^®^ vaccination. At the end of the study period there were 16 live and 22 dead dogs; median survival time of the live dogs was 29 months (range 2–46 months) versus 8 months (range 2–16 months) for those that died from progressive disease. This study showed that by using a combination of surgical excision and vaccination with Oncept^®^ survival times in dogs with malignant melanoma of the oral cavity, digit and other sites can be increased significantly.

## Introduction

Melanoma is a spontaneously occurring, highly aggressive and frequently metastatic neoplasm that affects the oral cavity, nail bed, footpad, mucocutaneous junctions and skin (Bergman *et al*. 2006). It is a relatively common diagnosis, representing approximately 4% of all canine tumours (MacEwen *et al*. 1986). It is also the most common oral tumour and second most common tumour affecting the digit in the dog (Henry *et al*. 2005; MacEwen *et al*. 1986; Marino *et al*. 1995). Treatment options for melanoma include surgery, chemotherapy, radiation, intralesional therapy and immunotherapy. However, local recurrence and distant metastasis are still frequent despite treatment (Bergman *et al*. 2006).

Dogs with advanced disease (World Health Organization stage II–IV) have a reported median survival time (MST) of 1–5 months with aggressive local excision (Bostock 1979; Harvey *et al*. 1981; MacEwen *et al*. 1986). Digital melanomas treated with surgery have a reported MST of 12 months, with approximately 50% of cases alive after 1 year and 13% alive after 2 years (Henry *et al*. 2005; Marino *et al*. 1995; Wobeser *et al.* 2007). A combination of hypo-fractionated radiation therapy and chemotherapy has a reported MST of one year in stage I oral melanoma (Freeman *et al*. 2003). Response rates to chemotherapy in melanoma range from 8% to 28%, with little evidence that treatment improves survival (Chapman *et al*. 1999; Rassnick *et al*. 2001). A study evaluating the efficacy of intralesional cisplatin treatment for malignant melanoma reported a partial to complete response in 14 of 20 dogs treated (Kitchell *et al*. 1994).

The concept of vaccinating against tumours dates back to 1893, where the observation that spontaneous regression of sarcomas occurred in humans with acute bacterial infections led to the hypothesis that the bacterial infection stimulated the immune system, which was then able to mount a response to destroy the tumour (Bergman & Wolchok 2008). Using the human tyrosinase gene a xenogeneic DNA vaccine was developed for use in dogs (Oncept^®^, 2010 Merial Limited, Duluth, GA). The vaccine contains human tyrosinase, a melanosomal glycoprotein that is essential in melanin synthesis. The tyrosinase antigen is transcribed and translated in the canine host and is recognised and processed in the context of its relevant major histocompatibility complex and associated co-stimulatory molecules (Grosenbaugh *et al*. 2011). It has also been shown that antigen-specific interferon-γ T-cell responses in dogs are potentiated by delivery of the vaccine through a needle-free transdermal delivery device (Goubier *et al*. 2008).

Oncept^®^ is registered for the management of dogs with stage II or III oral melanoma after local control has been achieved. The vaccine contains 102 μg of human tyrosine DNA per injection and is administered intramuscularly into the medial thigh region with a transdermal device. In initial studies one dog with stage IV disease had a complete clinical response in multiple lung metastases for 329 days; two dogs with stage IV disease had long-term survivals (421 and 588 days respectively) in the face of significant bulky metastatic disease, and two other dogs with locally controlled stage II/III disease had long-term survivals (501 and 496 days respectively), with no evidence of melanoma on necropsy (Bergman *et al*. 2003). Four other dogs were euthanased because of progression of the primary tumour (Bergman *et al*. 2003). The median survival time was 389 days (Bergman *et al*. 2003).

Further studies have shown that in dogs with oral melanoma survival time until death attributable to tumour has been significantly improved in those that received the vaccine compared with that of historical controls: 464 versus 156 days respectively (Grosenbaugh *et al*. 2011). In contrast, a more recent independent retrospective study examining the efficacy of this vaccine for the adjunct treatment of oral melanoma did not provide any evidence that vaccination affected the outcome of dogs with oral melanoma where loco-regional disease control had been achieved (Ottnod *et al*. 2013). This study showed that dogs that received the vaccine did not achieve a greater progression-free survival, disease-free interval or MST than historical controls.

Another recent study looking at the efficacy of the murine tyrosinase DNA vaccine in dogs with digit melanoma suggested prolongation of survival in the vaccinated dogs compared with historical controls treated with surgery alone (MST of 476 days in the treated vs 365 days in the historical controls) (Manley *et al*. 2011).

The purpose of this retrospective study was to document the investigators’ experiences with the Oncept^®^ vaccine when used as an adjunct therapy for treatment of stage II–IV oral, digit and malignant melanoma of other sites after local control had been achieved in 38 dogs presented to a South African specialist referral veterinary practice.

## Materials and methods

The medical records of dogs that were diagnosed with melanoma between March 2009 and July 2014 were evaluated retrospectively. Inclusion criteria for the study were a histopathological diagnosis of melanoma, surgical excision of the tumour, and a complete initial induction vaccination course using Oncept^®^.

Information obtained from the medical records included signalment, tumour location (oral vs digit vs other sites), clinical disease stage (based on standard World Health Organization guidelines for both digit and oral tumours), type of tumour (melanotic vs amelanotic), date of surgical excision and completeness of excision, date/cause of death or euthanasia, and follow-up schedule. Dogs were evaluated for metastatic disease via three-view thoracic radiography and cytological examination of fine-needle aspirate samples or histological evaluation of regional lymph node biopsy samples. MST was defined as the date from surgical excision to the date of euthanasia/death or the end date of the study.

The Oncept^®^ vaccine was administered according to the manufacturer’s instructions, namely four vaccine doses at two-weekly intervals using a transdermal injector device. Surviving dogs were administered booster injections at six-monthly intervals if agreed to by the owner.

All dogs were monitored for acute post-vaccination reactions (e.g. anaphylaxis, signs of pain or wheal formation) for just over 30 minutes after vaccine administration. At each subsequent vaccination injection sites were re-examined by the same investigators for evidence of residual injection-site reactions. Owners were requested to bring their dogs to the investigators or to notify the investigators if any adverse effects were detected at home.

## Results

A total of 38 dogs met the inclusion criteria, of which 25 dogs had oral, 6 digit, and 7 infiltrative cutaneous melanoma in other sites.

### Oral melanoma

There were 25 stage II–IV cases (23 stage II, 1 stage III and 1 stage IV) with a median age of 10 years (range 5–14). Of the 25 dogs, 3 (12%) had a histopathological diagnosis of amelanotic melanoma. Clear surgical margins were histopathologically confirmed in 15 of the 25 dogs (60%). At the end of the study there were 6 alive with a MST of 26 months (range 2–46 months) and 19 dead – 16 with progressive disease and the other 3 from unrelated causes (1 case each of gastric torsion, severe degenerative joint disease and osteosarcoma of the proximal humerus). The MST of the dogs that died as a result of progressive disease was 11.5 months (range 5–24 months). The MST of dogs that died as a result of progressive disease where surgical margins were found to be complete was 12 months (range 5–24 months) versus 7 months (range 6–16 months) in those where surgical margins were found to be incomplete. The MST of dogs that died with progressive disease in confirmed cases of amelanotic melanoma was 7 months versus 11 months (range 5–16 months) for those with confirmed melanotic melanomas. Sex distribution was 12 males and 13 females of various breeds: dachshund (4), German shepherd dog (3), spaniel (3), pekinese (2), Staffordshire terrier (2) and bouvier, giant schnauzer, maltese, Irish setter, Kerry blue terrier, golden retriever, Scottish terrier, rottweiler, doberman, crossbreed and great dane (1 of each).

### Digit melanoma

There were six stage II cases with an equal sex distribution and a median age of 8.5 years (range 3–10 years). All six dogs had a histopathological diagnosis of melanotic melanoma with confirmed clear surgical margins. At the end of the study five were still alive and one was dead, the latter following surgery for resection of a rib osteosarcoma. Breeds affected were golden retriever (2), bouvier (1), chow (1), miniature schnauzer (1) and Shar Pei (1). The MST of the five survivors was 36 months (range 11–46 months). Survival time of the dog that died was 12 months.

### Other sites

There were seven stage II–IV (two stage II, three stage III and two stage IV) cases with a sex distribution of four females and three males and a median age of 10 years (range 1–11 years). One dog had a histopathological diagnosis of amelanotic melanoma whilst clear surgical margins were histopathologically confirmed in three of the seven dogs (42%). Anatomical regions involved included skin in the inguinal area, ventral abdomen, axilla, forelimb, hind limb, between the eyes and lateral thorax. Affected breeds were dachshund (3), Staffordshire terrier (2), ridgeback (1) and golden retriever (1). MST of the five survivors was 22 months (range 4–45 months). Survival times of the two dogs that died were 2 and 6 months; both had histopathologically confirmed stage III melanotic melanomas with incomplete surgical margins.

### Overall outcome

Of the 38 dogs in this study, 16 are still alive and 22 are dead. The MST of the dogs which are still alive was 29 months (range 2–46 months) versus 8 months (range 2–16 months) in those that died of progressive disease. None of the dogs showed any adverse effects due to the vaccine.

## Discussion

The purpose of this retrospective study was to document the investigators’ experiences with the Oncept^®^ vaccine when used as an adjunct therapy for treatment of stage II–IV oral, digit and malignant melanoma of other sites after local control had been achieved in 38 dogs presented to a South African specialist referral veterinary practice. Dogs were divided into three groups based on anatomical location of the tumour (oral vs digit vs other sites) and were evaluated separately.

In terms of the group of dogs with oral melanoma, 24% were still alive at the end of the study, with a MST of 26 months (806 days). This is greater than reported in another study, where only 15% were alive at the end of the study (8 of 58 dogs) (Grosenbaugh *et al*. 2011). Of the dogs that died, 12% of the deaths were attributed to causes unrelated to disease progression. The MST of those dogs that died from progressive disease was 11.5 months (357 days), which is similar to the reported MST of historical controls in another similar study (324 days) (Grosenbaugh *et al*. 2011).

In dogs with oral melanoma there also appeared to be some survival advantage of a diagnosis of melanotic melanoma versus amelanotic melanoma (MST of 341 days vs 217 days) and the presence of complete surgical excision versus incomplete surgical excision (MST of 372 days vs 217 days). These results may, however, be compromised by the fact that both dogs with stage III and IV disease were in the incomplete excision group and none of the amelanotic melanomas were completely surgically excised.

In terms of the group of dogs with digit melanoma, at the end of the study period 83% were still alive, with a median survival time of 36 months (1116 days), whilst none of dogs in this group died as a result of progression of the disease. This survival time is remarkably longer than that reported in historical controls treated with surgery alone (MST 365 days) (Marino *et al*. 1995). Similar to previous reports, dogs diagnosed with digit melanoma in this study were all primarily large breeds and the majority were > 20 kg (Henry *et al*. 2005; Manley *et al*. 2011). Unlike in previous studies, in this study the hind limbs were more frequently affected than the fore limbs (Henry *et al*. 2005; Manley *et al*. 2011; Wobeser *et al.* 2007).

In terms of the group of dogs with malignant melanoma of other sites, at the end of the study period 71% were still alive, with a MST of 22 months (682 days). Both dogs that succumbed to the disease (survival times of 62 and 186 days respectively) had more aggressive, infiltrative, stage III melanomas that were incompletely surgically excised. This survival time is significantly longer than that reported by Bostock (1979) for cutaneous melanoma with malignant criteria treated with surgical excision alone, where 45% of dogs died within 1 year and 47% within 2 years of surgery. A more recent study included both benign and malignant melanomas treated with surgery alone (Spangler & Kass 2006); only 39% of cutaneous submissions were reported as histologically malignant, with only 12% recurring or metastasising and only 7% of dogs dying from tumour-related causes. A MST of almost 2 years (725 days) was reported for dogs with a cutaneous location (Spangler & Kass 2006).

Although the Oncept^®^ vaccine is only registered for use as an adjunct treatment for oral melanoma, this study suggests that there are definite survival advantages when it is also used in digit and malignant melanoma of other sites when local control has been achieved.

In all three groups no adverse effects (local or systemic) due to vaccination were noted by the investigators or reported by owners throughout the study period.

## Limitations of the study

Limitations of this study include its retrospective nature and the relatively small numbers of animals within the digit and other sites groups.

## Conclusion

This study showed that using a combination of surgical excision and vaccination with Oncept^®^ can significantly increase survival times in dogs with malignant melanoma of the oral cavity, digit and other sites.
